# Inconclusive effects between executive functions and symptoms of psychiatric disorders in random-intercept cross-lagged panel models: a simulated reanalysis and comment on Halse et al. (2022)

**DOI:** 10.3389/fpsyg.2025.1500200

**Published:** 2025-02-28

**Authors:** Kimmo Sorjonen, Bo Melin

**Affiliations:** Department of Clinical Neuroscience, Karolinska Institutet, Stockholm, Sweden

**Keywords:** executive deficits, random-intercept cross-lagged panel models, reanalyses, spurious effects, symptoms of psychopathology, triangulation

## Abstract

**Background:**

In a recent study of Norwegian children (*N* = 874), Halse et al. used random-intercept cross-lagged panel models (RI-CLPM) and concluded that their findings supported the assumption that deficiencies in executive functions and psychopathology are both a cause and a consequence of the other. However, it is known that RI-CLPM can give biased results.

**Methods:**

We reanalyzed data simulated to resemble the data used by Halse et al. with several complementary models, e.g., latent change score models (LCSM).

**Results:**

The analyzed models indicated contradictory simultaneous increasing and decreasing effects between executive deficits and symptoms of psychopathology.

**Conclusion:**

The present contradictory findings suggested that prospective effects between executive deficits and symptoms of psychopathology may have been spurious rather than truly increasing. Consequently, conclusions by Halse et al. appear to have been premature. It is important for researchers to bear in mind that correlations, including cross-lagged effects in RI-CLPM, do not prove causality. Careful interpretation of RI-CLPM results is of utmost importance in, for example, research in clinical and developmental psychology. We recommend researchers to use, as we did here, triangulation to scrutinize findings from analyses of observational data.

## Introduction

In random-intercept cross-lagged panel models (RI-CLPM), longitudinally measured scores on two (or more) constructs are regressed on stable, trait-like, latent variables (the random intercepts). Then, auto-regressive and cross-lagged effects are estimated between residuals of the measured scores not accounted for by individuals’ stable levels. These residuals could, for example, be occasion-specific deviations from individuals’ stable levels of anxiety and depression and the cross-lagged effects would estimate how deviation in anxiety at time T predicts deviation in depression at time T + 1 when adjusting for deviation in depression at time T and vice versa. In this way, effects are presumably estimated within individuals (Hamaker et al., 2015; Mulder and Hamaker, 2021). Within-individual effects should give better estimations of true increasing or decreasing effects compared with effects in traditional cross-lagged panel models (CLPM), where effects are conflated by between-individual differences (Usami et al., 2019).

Halse et al. (2022), used RI-CLPM and concluded that their findings supported the assumption that deficiencies in executive functions and psychopathology are both a cause and a consequence of the other. However, there are indications that RI-CLPM, similarly as CLPM, may be susceptible to spurious findings (Lüdtke and Robitzsch, 2021; Lucas, 2023; Sorjonen et al., 2023; Murayama and Gfrörer, 2024). For example, if longitudinal scores on two constructs X and Y are affected by common auto-correlated state factors, RI-CLPM will tend to indicate statistically significant, but spurious, cross-lagged effects (Sorjonen et al., 2024b). For example, imagine some individuals with the same within-individual score on anxiety at time T (i.e., they deviate equally much from their stable levels of anxiety) but who differ in their within-individual score on depression at time T. In this situation, we should expect that those with a higher score on within-individual depression have received a lower score on within-individual anxiety than they should have (i.e., a more negative residual) while those with a lower within-individual score on depression have received a higher score than they should have (i.e., a more positive residual). However, residuals tend to regress toward a mean value of zero between measurements. Consequently, we should expect a more positive, but spurious, change in within-individual anxiety between time T and time T + 1 for those with a higher within-individual score on depression at time T compared with those with the same within-individual score on anxiety but a lower within-individual score on depression at time T. Consequently, positive cross-lagged effects between within-individual depression and within-individual anxiety in an analysis with RI-CLPM could be due to correlations with residuals and regression to the mean rather than genuine (i.e., non-spurious) increasing effects.

Given indications that results from RI-CLPM may be spurious, the objective of the present study was to reanalyze data simulated to resemble the data used by Halse et al. (2022) and to evaluate their conclusion of causal effects between deficiencies in executive functions and psychopathology. We used simulated data as the empirical data used by Halse et al. were not available to us. Our reanalyses followed recommendations to triangulate findings from analyses of correlational (i.e., non-experimental) data (Munafò and Davey Smith, 2018; Hammerton and Munafò, 2021; Sorjonen and Melin, 2024). If results from different models converge, conclusions of causality are corroborated. If, on the other hand, results from different models diverge, conclusions of causality would appear premature.

## Method

### Data

We refer to Halse et al. (2022) for more comprehensive information on the study sample, used instruments, research procedure, etc. In short, data on deficits in executive functions and symptoms of depression, oppositional defiant disorder and conduct disorder (ODD/CD), attention-deficit hyperactivity disorder (ADHD), and anxiety were collected for children (*N* = 874) born in Norway between 2003 and 2004. Data were collected on five occasions (every second year) when the children were between 6 and 14 years old.

We used simulated data as the empirical data used by Halse et al. were not available to us. It is important to bear in mind that RI-CLPM, as well as other types of structural equation models (SEM), extract covariances or correlations (for standardized parameters) from raw data and use these as input for the analyses. Consequently, although slight deviations can be expected due to missing values and non-normality in empirical data, analyses of simulated data should yield very similar results as analyses of empirical data. For example, a cross-lagged effect of X1 on Y2 when adjusting for Y1 is given by correlations between the variables (Equation 1, Cohen et al., 2003). This means that the cross-lagged effect would be the same in simulated data with the same correlations between variables as in empirical data.


(1) $$E|\beta_{X,Y2.Y1}|=\frac{r_{X,Y2}-r_{X,Y1}r_{Y1,Y2}}{1-r_{X,Y1}^{2}}$$

### Analyses

**Figure 1:**
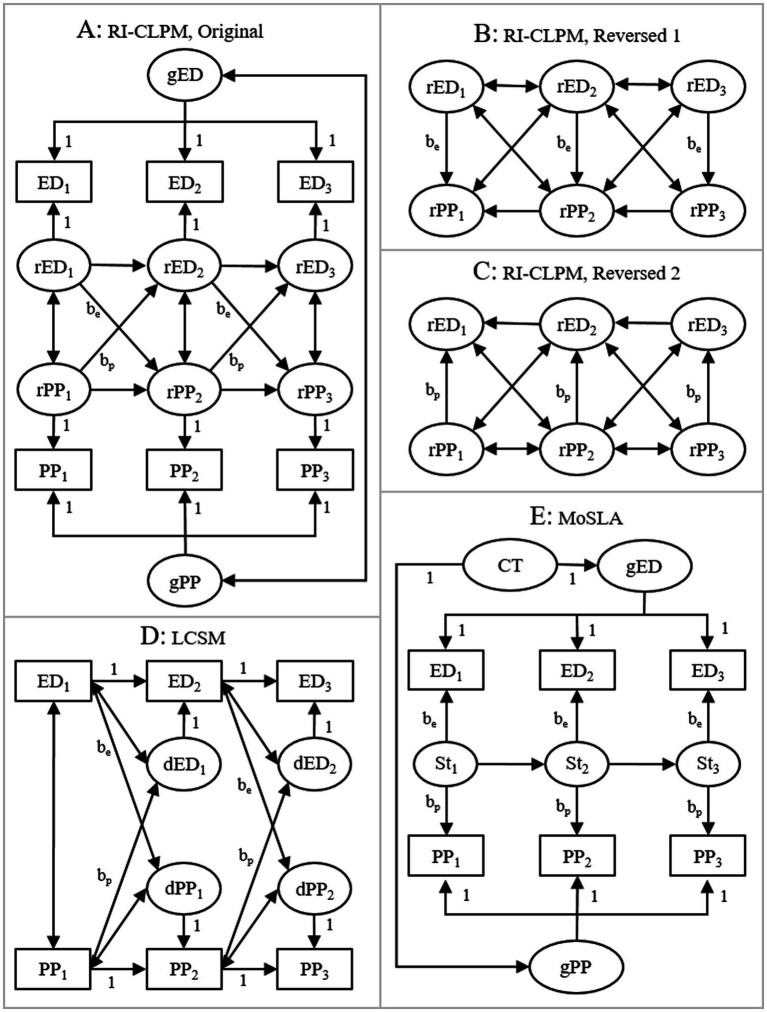
The five analyzed models. **(A)** Original RI-CLPM, where initial within-individual levels of executive deficits predicted subsequent within-individual levels of psychopathology when adjusting for initial within-individual levels of psychopathology and vice versa; **(B)** Reversed RI-CLPM, where initial within-individual levels of executive deficits predicted initial within-individual levels of psychopathology when adjusting for subsequent within-individual levels of psychopathology. Parameters between random intercepts and the measured scores not shown; **(C)** A second reversed RI-CLPM, where initial within-individual levels of psychopathology predicted initial within-individual levels of executive deficits when adjusting for subsequent within-individual levels of executive deficits. Parameters between random intercepts and the measured scores not shown; **(D)** Latent change score model (LCSM), where initial levels of executive deficits predicted subsequent latent change in levels of psychopathology and vice versa; **(E)** Model of spurious longitudinal associations (MoSLA), where scores on executive deficits and psychopathology were regressed on general, trait-like, random intercepts as well as on auto-correlated state factors. ED, executive deficits; PP, psychopathology; CT, common trait-factor; 1, 2, 3 = measurements at waves 1, 2, and 3, respectively; gED/gPP, general, trait-like, levels (i.e., random intercepts) of executive deficits and psychopathology, respectively; rED/rPP, within-individual residuals of executive deficits and psychopathology, respectively; dED/dPP, latent change in executive deficits and psychopathology, respectively; St, state factor; *b_e_*/*b_p_*, effects of main interest; analyzed data contained five waves of measurement rather than three as illustrated here.

We fitted five different models (Figure 1) on the simulated data, separately for each of the four measures of psychopathology: (A) Original RI-CLPM, where within-individual residuals of executive deficits at time T predicted within-individual residuals of symptoms of psychopathology at time T + 1 when adjusting for within-individual residuals of symptoms of psychopathology at time T, and vice versa; (B) Reversed RI-CLPM, where within-individual residuals of executive deficits at time T predicted within-individual residuals of symptoms of psychopathology at time T when adjusting for within-individual residuals of symptoms of psychopathology at time T + 1; (C) A second reversed RI-CLPM, where within-individual residuals of symptoms of psychopathology at time T predicted within-individual residuals of executive deficits at time T when adjusting for within-individual residuals of executive deficits at time T + 1; (D) Latent change score model (LCSM) (McArdle, 2009; Ghisletta and McArdle, 2012; Kievit et al., 2018), where executive deficits at time T predicted latent change in symptoms of psychopathology between times T and T + 1 and vice versa; (E) Model of spurious longitudinal associations (MoSLA) (Sorjonen and Melin, 2024), where the five measurements of executive deficits and symptoms of psychopathology were regressed on stable trait-like levels of the constructs, i.e., random intercepts, as well as on auto-correlated state factors affecting executive deficits and psychopathology measured at the same occasion. The trait-like levels of executive deficits and psychopathology were affected, i.e., confounded, by a common trait-factor. Analyses were conducted with R 4.3.1 statistical software (R Core Team, 2024) employing the lavaan (Rosseel, 2012) and MASS (Venables and Ripley, 2002) packages. The analytic script, which also generates the simulated data, is available at the Open Science Framework at https://osf.io/fs8c7/.

### Predictions

If executive deficits and psychopathology had, as concluded by Halse et al. (2022), causal increasing effects on each other, we should expect focal effects (labeled *b_e_* and *b_p_* in Figure 1) to be positive in models A and D but negative in models B and C. Although negative effects may appear counterintuitive, they would indicate that a low initial within-individual value on executive deficits had compensated for a high initial within-individual value on psychopathology and allowed individuals to reach the same subsequent within-individual level of psychopathology as individuals with a lower initial within-individual value on psychopathology but a higher initial within-individual value on executive deficits, and vice versa. For example, an effect of −0.5 of initial within-individual executive deficits on initial within-individual psychopathology when adjusting for subsequent within-individual psychopathology would mean that among individuals with the same subsequent within-individual level of psychopathology (e.g., zero), those with lower initial within-individual executive deficits (e.g., −1) had had a higher initial within-individual level of psychopathology (−1 × −0.5 = 0.5) compared with those with higher initial within-individual executive deficits (e.g., 1 and 1 × −0.5 = −0.5). This would, in turn, mean that those with lower initial within-individual executive deficits had experienced a more negative change in within-individual psychopathology between the measurements (0–0.5 = −0.5) compared with those with the same subsequent within-individual level of psychopathology but with a higher initial within-individual level of executive deficits (0 – (−0.5) = 0.5). As an analogy, given that pouring water into a glass has an increasing effect on its total weight (i.e., including its content), if we pour different amounts of water into glasses that then have the same subsequent weight, those glasses that received least water should have had the highest initial weight. This corresponds to a negative effect of the amount of poured water on initial weight when adjusting for subsequent weight and would indicate that a high initial weight had compensated for receiving little water and allowed the glass to have the same subsequent weight as glasses that received more water. Moreover, an adequate fit of model E would indicate that data may have been generated without any genuine effects between executive deficits and psychopathology, i.e., the apparent increasing effects in the RI-CLPM may have been spurious.

## Results

The size of focal effects (labeled *b_e_* and *b_p_* in Figure 1) as well as model fits are presented in Table 1. With the possible exception of LCSM (model D), the models exhibited adequate fit for all four measures of symptoms of psychopathology. In the original RI-CLPM (model A), the positive focal effects indicated that when adjusting for initial within-individual levels of psychopathology, initial within-individual levels of executive deficits had an increasing effect on subsequent change in within-individual levels of psychopathology and vice versa. However, the positive focal effects in the two reversed RI-CLPMs (models B and C) indicated contradictory decreasing effects between within-individual levels of executive deficits and psychopathology. For example, among individuals with the same subsequent within-individual level of depression (e.g., zero), those with a low initial within-individual level of executive deficits (e.g., −1) were predicted to have had a lower initial within-individual level of depression (−1 × 0.13 = −0.13) compared with those with a high initial within-individual level of executive deficits (e.g., 1 and 1 × 0.13 = 0.13). Consequently, those with a low initial within-individual level of executive deficits were predicted to have increased in within-individual level of depression between measurements (0 – (−0.13) = 0.13) while those with a high initial within-individual level of executive deficits were predicted to have decreased in within-individual level of depression between measurements (0–0.13 = −0.13). The positive focal effects in models B and C suggest that high, not low, initial within-individual levels of executive deficits had compensated for high initial within-individual levels of psychopathology and allowed individuals to reach the same subsequent within-individual levels of psychopathology as individuals with lower initial within-individual levels of executive deficits and psychopathology and vice versa. Moreover, the LCSMs (model D) did not indicate increasing effects of initial executive deficits on subsequent change in symptoms of psychopathology and vice versa. Rather, these effects tended to be negative. Furthermore, the adequate fit of the MoSLA (model E) suggested that data may have been generated without any genuine effects between executive deficits and symptoms of psychopathology, i.e., the apparent increasing effects in the RI-CLPM may have been spurious. In summary, conclusions of reciprocal prospective increasing effects between executive deficits and symptoms of psychopathology were supported by the original RI-CLPM (Model A) but contested by the other models (Models B–E).

**Table 1:** Effects between deficits in executive functions and symptoms of psychiatric disorders, separately for four different disorders and five different models.

Out/Model	*b_e_* [95% CI]	*b_p_* [95% CI]	χ^2^	df	CFI	TLI	RMSEA [90% CI]
Depression							
A (org.)	0.15 [0.10; 0.20]	0.11 [0.07; 0.15]	168	43	0.962	0.960	0.058 [0.049; 0.067]
B (rev1.)	0.13 [0.09; 0.17]	-	247	46	0.939	0.940	0.071 [0.062; 0.079]
C (rev2.)	-	0.10 [0.07; 0.14]	236	46	0.942	0.943	0.069 [0.060; 0.077]
D (LCSM)	−0.02 [−0.03; 0.00]	−0.02 [−0.05; 0.00]	446	56	0.881	0.904	0.089 [0.082; 0.097]
E (MoSLA)	0.60 [0.54; 0.67]	0.12 [0.09; 0.16]	278	49	0.930	0.936	0.073 [0.065; 0.082]
ODD/CD							
A (org.)	0.23 [0.18; 0.27]	0.15 [0.10; 0.19]	101	43	0.985	0.984	0.039 [0.029; 0.049]
B (rev1.)	0.15 [0.11; 0.19]	-	184	46	0.963	0.964	0.059 [0.050; 0.068]
C (rev2.)	-	0.14 [0.10; 0.18]	193	46	0.961	0.962	0.061 [0.052; 0.069]
D (LCSM)	−0.02 [−0.04; 0.00]	−0.03 [−0.05; −0.01]	410	56	0.906	0.925	0.085 [0.077; 0.093]
E (MoSLA)	0.54 [0.49; 0.60]	0.17 [0.14; 0.21]	217	49	0.956	0.959	0.063 [0.054; 0.071]
ADHD							
A (org.)	0.18 [0.13; 0.22]	0.02 [−0.02; 0.07]	163	43	0.977	0.976	0.057 [0.048; 0.066]
B (rev1.)	0.15 [0.11; 0.19]	-	218	46	0.967	0.968	0.065 [0.057; 0.074]
C (rev2.)	-	0.21 [0.17; 0.25]	232	46	0.964	0.965	0.068 [0.059; 0.077]
D (LCSM)	−0.01 [−0.03; 0.02]	−0.07 [−0.09; −0.05]	507	56	0.913	0.930	0.096 [0.088; 0.104]
E (MoSLA)	0.58 [0.52; 0.65]	0.14 [0.11; 0.17]	338	49	0.944	0.949	0.082 [0.074; 0.091]
Anxiety							
A (org.)	0.16 [0.11; 0.21]	0.10 [0.06; 0.14]	92	43	0.985	0.984	0.036 [0.026; 0.046]
B (rev1.)	0.06 [0.01; 0.10]	-	171	46	0.962	0.963	0.056 [0.047; 0.065]
C (rev2.)	-	0.05 [0.01; 0.08]	172	46	0.962	0.963	0.056 [0.047; 0.065]
D (LCSM)	0.00 [−0.02; 0.02]	−0.01 [−0.04; 0.01]	387	56	0.899	0.919	0.082 [0.075; 0.090]
E (MoSLA)	0.61 [0.54; 0.68]	0.09 [0.06; 0.12]	224	49	0.947	0.951	0.064 [0.056; 0.073]

See Figure 1 for illustrations of the models and which effects *b_e_* and *b_p_* refer to.

## Discussion

The present study set out to conduct reanalyses and to evaluate the conclusion by Halse et al. (2022) of causal effects between deficiencies in executive functions and psychopathology. Random-intercept cross-lagged panel models (RI-CLPM) indicated, in agreement with Halse et al., increasing effects between within-individual levels of executive deficits and symptoms of psychopathology. However, reversed RI-CLPMs and latent change score models (LCSM) indicated contradictory decreasing effects between executive deficits and symptoms of psychopathology. Moreover, models of spurious longitudinal associations (MoSLAs) indicated that data may have been generated without any genuine effects between executive deficits and symptoms of psychopathology, i.e., the apparent increasing effects in the RI-CLPM may have been spurious. Taken together, results from the present reanalyses and triangulation suggested that data analyzed by us and by Halse et al. do not support conclusions of causal effects between executive deficits and symptoms of psychopathology and, consequently, that the conclusions by Halse et al. in this regard were premature. In extension, the present findings also suggest that trying to alleviate symptoms of psychopathology through measures targeting executive deficits, and vice versa, may not be the best use of limited clinical resources.

The present reanalyses followed recommendations to triangulate findings from analyses of observational data (Munafò and Davey Smith, 2018; Hammerton and Munafò, 2021; Sorjonen and Melin, 2024). We agree with this recommendation, because correlations do not prove causality. This is true both of zero-order correlations and more advanced correlations like adjusted cross-lagged effects in traditional CLPM and in RI-CLPM. With longitudinal data, triangulations may utilize the fact that if a variable X (e.g., amount of water poured into a glass) has an increasing effect on a variable Y (e.g., the total weight of the glass), X should also compensate for a low initial value on Y. This means that with a genuine increasing effect of X on Y, X should have a positive effect on subsequent Y when adjusting for initial Y, a positive effect on the subsequent Y - initial Y difference, as well as a negative effect on initial Y when adjusting for subsequent Y. Without this combination of expected effects, observed effects would appear to be spurious and conclusions of causality premature. We (Sorjonen et al., 2023, 2024a, 2024b; Sorjonen and Melin, 2024) have previously used this type of triangulation and challenged conclusions in studies using RI-CLPM and claiming effects of need of cognition on anxiety and depression symptoms (Zainal and Newman, 2022), of income on self-esteem (Bleidorn et al., 2023), of curiosity on creativity and vice versa (Ma and Wei, 2023), and of self-esteem on eating pathology and vice versa (Beckers et al., 2023). We recommend researchers to use similar triangulations to scrutinize findings from analyses of observational data.

### Limitations

As we did not have access to the empirical data used by Halse et al. (2022), we reanalyzed data simulated to have the same sample size and correlations between variables as in their empirical dataset. Some may view this as a major limitation of the present study. However, it is important to bear in mind that RI-CLPM, as well as other types of structural equation models (SEM), extract covariances or correlations (for standardized parameters) from raw data and use these as input for the analyses. Consequently, although slight deviations can be expected due to missing values and non-normality in empirical data, analyses of simulated data should yield very similar results as analyses of empirical data. In the present case, this conclusion was corroborated by the fact that original RI-CLPMs indicated, in agreement with Halse et al., increasing effects between executive deficits and symptoms of psychopathology. The crucial difference between our analyses and analyses by Halse et al. was not that we used simulated data, but that we, differently from them, used triangulation and fitted several complementary models to the same data, which allowed us to discriminate between genuine and spurious increasing effects. Moreover, reliability is a function of correlations. Consequently, as the correlations in our simulated dataset were the same as in the original empirical dataset used by Halse et al., there were no differences in measurement reliability between the two datasets.

The data collection may not have utilized optimal instruments, study procedures, etc. However, it is, again, important to bear in mind that such factors were constant across the analyzed models, meaning that they cannot explain why the models indicated simultaneous and contradictory increasing and decreasing effects between executive deficits and symptoms of psychopathology. Consequently, such possible deficiencies do not pose a threat against our conclusion that the data analyzed by Halse et al., and reanalyzed by us, do not allow conclusions of causal increasing effects between executive deficits and symptoms of psychopathology.

The present study was based on data simulating data collected in a, presumably quite homogenous, sample of Norwegian children. It is unclear to what degree the present main finding, that prospective effects between executive deficits and symptoms of psychopathology appear to be spurious rather than genuinely increasing, generalizes to other populations. It should be noted that a potential limitation in generalizability is due to where and how the original data were collected and not a graver threat against our study employing simulated data than against the original study by Halse et al.

## Conclusion

Halse et al. (2022) conducted analyses with random-intercept cross-lagged panel models (RI-CLPM) and concluded that their findings supported the assumption that deficiencies in executive functions and psychopathology are both a cause and a consequence of the other. Here, we reanalyzed data simulated to resemble the data used by Halse et al. Our findings suggested that prospective effects between executive deficits and symptoms of psychopathology may have been spurious rather than truly increasing. Consequently, conclusions by Halse et al. appear to have been premature. It is important for researchers to bear in mind that correlations, including cross-lagged effects in RI-CLPM, do not prove causality. We recommend researchers to use, as we did here, triangulation to scrutinize findings from analyses of observational data.

## Data Availability

The datasets presented in this study can be found in online repositories. The names of the repository/repositories and accession number(s) can be found below: the analytic script, which also generates the simulated data, is available at the Open Science Framework at https://osf.io/fs8c7/.
